# Clinical Implications of Aberrant PD-1 and CTLA4 Expression for Cancer Immunity and Prognosis: A Pan-Cancer Study

**DOI:** 10.3389/fimmu.2020.02048

**Published:** 2020-09-10

**Authors:** Jian-Nan Liu, Xiang-Shuo Kong, Tao Huang, Rui Wang, Wang Li, Qi-Feng Chen

**Affiliations:** ^1^Department of Oncology, Yantai Yuhuangding Hospital, Yantai, China; ^2^Department of Medical Imaging and Interventional Radiology, Sun Yat-sen University Cancer Center, Guangzhou, China; ^3^State Key Laboratory of Oncology in South China, Guangzhou, China; ^4^Collaborative Innovation Center for Cancer Medicine, Guangzhou, Guangdong, China; ^5^Department of Respiratory Oncology, Fushan District People's Hospital, Yantai, China

**Keywords:** pan-cancer, PD-1, CTLA4, prognostic biomarker, cancer immunity

## Abstract

Combination therapy with inhibitors of cytotoxic T lymphocyte-associated protein (CTLA)4 and programmed death (PD)-1 has demonstrated efficacy in cancer patients. However, there is little information on CTLA4 and PD-1 expression levels and their clinical significance across diverse cancers. In this study, we addressed this question by analyzing PD-1 and CTLA4 levels in 33 different types of cancer along with their prognostic significance using The Cancer Genome Atlas (TCGA) and Cancer Cell Line Encyclopedia datasets. Liver hepatocellular carcinoma (LIHC) patients receiving cytokine-induced killer cell (CIK) immunotherapy at Sun Yat-sen University cancer center were enrolled for survival analysis. The correlation between PD-1/CTLA4 expression and cancer immunity was also analyzed. The results showed that PD-1 and CTLA4 transcript levels varied across cancer cell lines, with aberrant expression detected in certain cancer types; Kaplan–Meier analysis with the Cox proportional hazards model showed that this was closely related to overall survival in breast invasive carcinoma, glioblastoma multiforme, head and neck squamous cell carcinoma, acute myeloid leukemialymphoma, uterine corpus endometrial carcinoma, and uveal melanoma in TCGA. High serum PD-1 and CTLA4 levels predicted better survival in LIHC patients receiving CIK therapy. PD-1 and CTLA4 levels were found to be significantly correlated with the degree of tumor cell infiltration using Tumor Immune Estimation Resource, Estimating Relative Subsets of RNA Transcripts, and Estimation of Stromal and immune Cells in Malignant Tumor Tissues Using Expression Data as well as with tumor-infiltrating lymphocyte marker expression; they were also related to tumor mutation burden, microsatellite instability, mismatch repair, and the expression of DNA methyltransferases in some cancer types. Gene set enrichment analysis of 33 cancer types provided further evidence for associations between PD-1/CTLA4 levels and cancer development and immunocyte infiltration. Thus, PD-1 and CTLA4 play important roles in tumorigenesis and tumor immunity and can serve as prognostic biomarkers in different cancer types.

## Introduction

Cancer is a leading cause of death worldwide, and the low efficacy of many existing therapies is a major clinical challenge (1). Molecular-level pan-cancer analyses have provided insights into the common features and heterogeneity of various human malignancies (2). For example, Cancer Cell Line Encyclopedia (CCLE) and The Cancer Genome Atlas (TCGA) were developed based on epigenomic, genomic, proteomic, and transcriptomic data from multiple human cancer cell lines and tissues (3–5). Pan-cancer analyses have also revealed the significance of specific genes and signaling pathways in cancers. For example, tumor hypoxia-associated multi-omic investigations have shown that some molecular variants are correlated with antitumor drug sensitivity or resistance, which has important implications for cancer treatment (6). The expression status of Forkhead box M1 and its relationship to etiology and outcomes of human cancers (7), as well as proteomic and genomic features related to MYC and the proximal MYC network (8) have been reported for 33 cancer types in TCGA. The expression of more than 9,000 genes in TCGA has been characterized in terms of their contribution to the immune phenotype of various cancers (9). Thus, pan-cancer analyses can be useful for the development of new combination treatments and individualized therapies.

The mechanisms of immune evasion by cancer cells are the target of immunotherapies (10). Cytotoxic T lymphocyte-associated protein (CTLA)4 and programmed death (PD)-1 are receptors that attenuate the T cell response (11). Both factors are the immune checkpoint inhibitors with distinct but complementary mechanisms of action. CTLA4 is a target for monoclonal antibody-based drugs that enhance anticancer immunity such as ipilimumab, which was the first CTLA4 inhibitor to be developed and the only one to date that has been approved by the U.S. Food and Drug Administration (FDA) (12). PD-1 is a transmembrane protein that is expressed by immunocytes; blocking PD-1 signaling enhances the anticancer effect of T cells, thereby promoting cancer cell killing. The combination of nivolumab (13)—which targets PD-1—and ipilimumab increased overall survival (OS) in patients with melanoma (14), renal cell carcinoma (15–17), and advanced non-small cell lung cancer (18), and has been approved for the treatment of hepatocellular carcinoma (LIHC) patients previously treated with sorafenib (19).

Although PD-1 and CTLA4 overexpression, mutations, and gene amplification have been reported in certain cancers, the studies had small sample sizes and used different experimental approaches, making it difficult to compare the findings. Additionally, these studies focused on a single or a few types of cancer; there have been no studies comparing multiple types of cancer. To this end, the present study investigated PD-1 and CTLA4 expression profiles and their prognostic significance in various human malignancies based on large CCLE and TCGA datasets. We also examined the associations between PD-1/CTLA4 expression and the extent of tumor cell infiltration, microsatellite instability (MSI), tumor mutational burden (TMB), DNA methyltransferase (DNMT) levels, and mismatch repair (MMR) in different tumor types by gene set enrichment analysis (GSEA). The results provide important insights into the roles of PD-1 and CTLA4 in anticancer immunity.

## Materials and Methods

### Patient Datasets and Processing

TCGA comprises over 20,000 samples from 33 types of cancer and corresponding non-carcinoma samples. Processed level 3 RNA sequencing data and corresponding clinical annotations were obtained from TCGA using the University of California Santa Cruz cancer genome browser (https://tcga.xenahubs.net; accessed April 2020). CCLE (https://portals.broadinstitute.org/ccle) provides genetic and pharmacologic information from a large number of human tumor models, with RNA sequencing data for over 1,000 cell lines. Ethics approval for use of human data was not required for this part of the study as only open-access datasets were used.

A total of 122 consecutive patients with LIHC (mean age, 46.8 years; range: 22–75 years) who underwent curative resection and received adjuvant cytokine-induced killer (CIK) cell immunotherapy at Sun Yat-sen University Cancer Center between March 2004 and January 2015 were enrolled. Preparation of CIK cells and the treatment schedule are described in our previous study (20). Preoperative patient serum samples were obtained from our hospital's sample bank and analyzed using an anti-PD-1 and -CTLA4 antibody array (RayBiotech, Norcross, GA, USA; product no. QAH-ICM-1-1) according to the manufacturer's instructions. Briefly, the array was incubated with blocking buffer for 1 h before 2-fold–diluted serum samples (60 μl) were added. After overnight incubation at 4°C followed by washes, biotin-conjugated detection antibody was added for 2 h. The array was washed and Alexa Fluor 555-conjugated streptavidin was added for 1 h at room temperature. An InnoScan 300 scanner (Inopsys, Carbonne, France) was used to detect the signals (532 nm excitation); raw data were processed as images and spot intensities using Mapix 7.3.1 software (Innopsys). Serum concentrations of PD-1 and CTLA4 proteins were determined by automatic normalization and calculation. Follow-up was conducted until November 2019, with a median time of 84.3 months (range: 11.6–134.7 months). The primary endpoint was OS. This study was approved by the Ethics Committee of Sun Yat-sen University Cancer Center. All participants provided written, informed consent. The analytical workflow is shown in Supplementary Figure 1.

### Correlation Between PD-1/CTLA4 Expression and Survival

Data on PD-1 and CTLA4 gene expression for 33 cancer types and adjacent non-carcinoma tissues were extracted from TCGA and used to generate an expression matrix, which was matched to clinical information by patient identification number. A univariate Cox proportional hazards model was used to calculate correlations between gene expression and patient survival, where *P* < 0.05 was taken as the threshold for a statistically significant difference for PD-1 and CTLA4 expression in a given cancer relative to normal tissue. A Kaplan–Meier survival analysis was carried out to compare OS of patients in TCGA, which was stratified according to median PD-1 and CTLA4 expression levels with the log-rank test.

### Relationship Between PD-1/CTLA4 and Tumor Immunity

Tumor Immune Estimation Resource (TIMER; https://cistrome.shinyapps.io/timer/) allows systemic analysis of immune infiltrates in different cancer types (21) using a deconvolution statistical approach to infer tumor-infiltrating lymphocyte (TIL) counts based on gene expression data (22). Using the TIMER algorithm, we examined the associations between PD-1/CTLA4 levels and the numbers of 6 immune infiltrates—namely, cluster of differentiation [CD]4+ T cells, CD8+ T cells, B cells, neutrophils, dendritic cells, and macrophages.

Estimating Relative Subsets of RNA Transcripts (CIBERSORT) is a metagene tool that can be used to predict the phenotypes of 22 human immunocytes, as previously reported for all TCGA samples (23). In this study, CIBERSORT was used to calculate the relative fractions of the 22 leukocyte types; the correlations between *PD-1/CTLA4 levels and each leukocyte across 33 cancer types was then determined*.

Estimation of Stromal and Immune Cells in Malignant Tumor Tissues Using Expression Data (ESTIMATE) uses gene expression profiles to predict the purity of a tumor based on infiltration of stromal cells/immunocytes (24). The ESTIMATE algorithm yields 3 scores based on GSEA of single samples, including (1) stromal score, which reflects the presence of stromal cells in tumor tissue; (2) immune score, which indicates the degree of immunocyte infiltration into tumor tissue; and (3) estimate score, which describes tumor purity. We used the algorithm to estimate both immune and stromal scores for a variety of tumor tissues, and evaluated the associations between the scores and PD-1/CTLA4 levels.

We also examined the associations between PD-1/CTLA4 levels and the expression of TIL markers (25–27). An expression heatmap was generated for gene pairs in specific cancer types and correlations were analyzed with Spearman's rank correlation test.

TMB measures the number of mutations in a specific cancer genome and is used as a biomarker to identify patients that are most likely to respond to checkpoint inhibitor therapy (28). We obtained somatic mutation data of all TCGA patients (https://tcga.xenahubs.net), calculated their TMB scores, and then analyzed the correlation between TMB and PD-1/CTLA4 level. MSI is characterized by length polymorphisms of microsatellite sequences resulting from DNA polymerase slippage. Patients with high MSI (MSI-H) cancers benefit from immunotherapy, and MSI is an index used for cancer detection (29). We computed the MSI score of each patient and performed a correlation analysis between MSI and PD-1/CTLA4. MMR, is a DNA repair mechanism in normal cells that corrects DNA replication errors. Gene mutation frequency may be increased in cancer cells as a result of downregulation of MMR genes or defective MMR (29). Here we analyzed the correlation between MMR gene (MutL homolog [MLH]1, MutS homolog [MSH]2, MSH6, postmeiotic segregation increased [PMS]2, and epithelial cell adhesion molecule [EPCAM]) and PD-1/CTLA4 expression levels. DNA methylation has been implicated in tumorigenesis and cancer progression. As DNMT1, DNMT2, DNMT3A, and DNMT3B are the major enzymes involved in DNA methylation (30), we analyzed the correlation between their expression levels and those of PD-1 and CTLA4.

### Functional Analysis

We carried out GSEA using the JAVA program (http://software.broadinstitute.org/gsea/index.jsp) to investigate the biological significance of PD-1 and CTLA4 expression levels in tumor tissues. The random sample permutation number was set as 100, and the threshold of significance was *P* < 0.05. The results were visualized with enrichment maps generated using Bioconductor (http://bioconductor.org/) and R v3.6.0 software (R Foundation, Vienna, Austria).

### Statistical Analysis

Survival was evaluated as OS (defined as the time from the date of diagnosis to death from any cause) and progression-free survival (PFS; defined as the time until disease progression or death from any cause). The Wilcox log-rank test was used to assess changes in the sum of gene expression z-scores of cancer tissues compared to adjacent normal tissues. Differences in PD-1 and CTLA4 levels between different tumor stages were compared with the Kruskal–Wallis test. Survival was analyzed with Kaplan–Meier curves, the log-rank test, and Cox proportional hazards regression model. Spearman's or Pearson's test was used for correlation analysis. All statistical analyses were performed using R software.

## Results

### Pan-Cancer Expression Profiles of PD-1 and CTLA4

**Figure 1:**
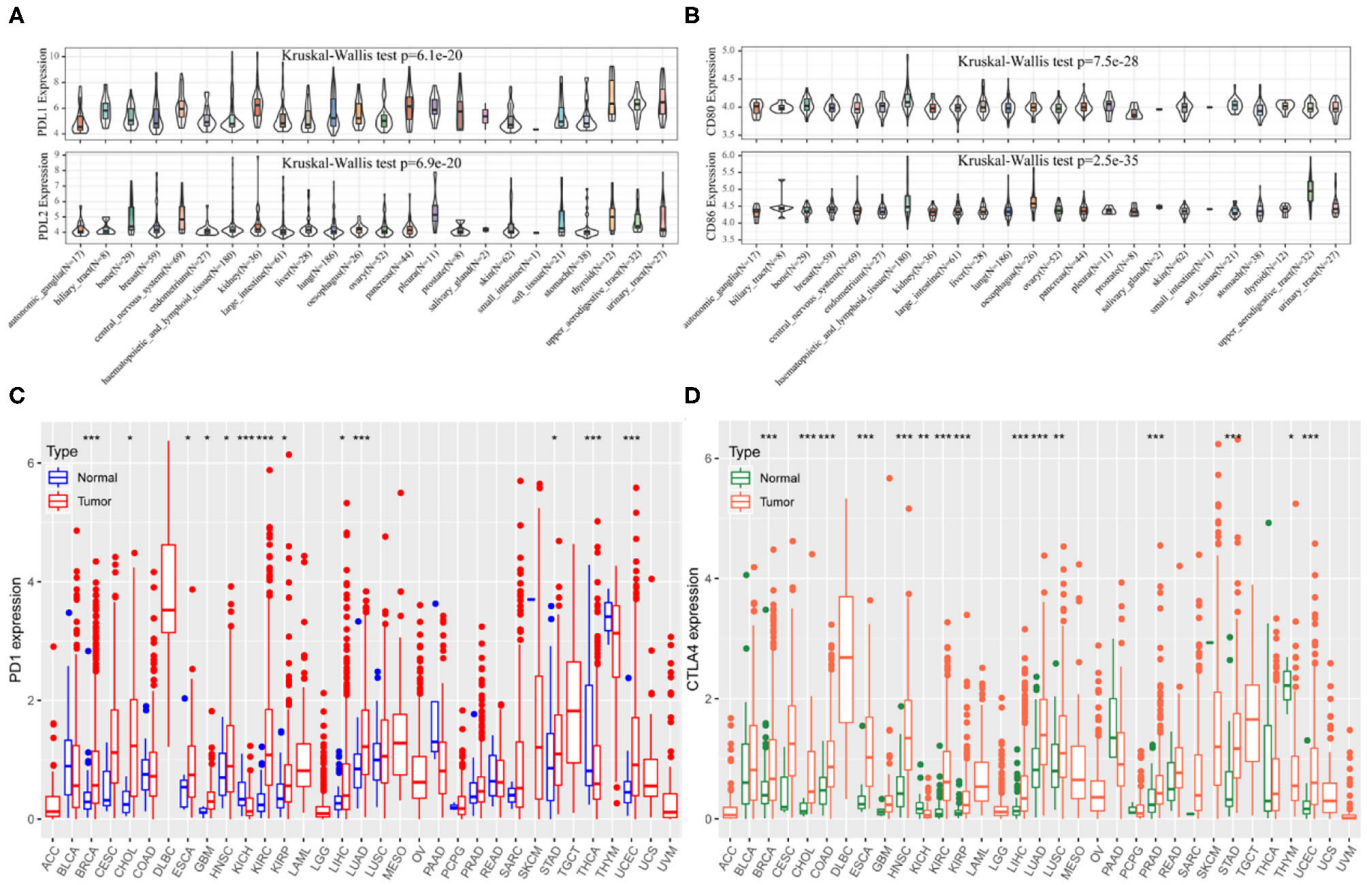
PD-1 and CTLA4 expression in different cancer types. **(A–D)** Expression levels of PD-1 **(A,C)** and CTLA4 **(B,D)** in CCLE **(A,B)** and TCGA **(C,D)** are shown. *P < 0.05, **P < 0.01, ***P < 0.001.

The CCLE data revealed variable expression of PD-1 and CTLA4 ligands across cancer cell lines (both *P* < 0.001; Figures 1A,B). Among the 33 cancer types in TCGA, PD-1 and CTLA4 levels were upregulated in tumor tissues relative to matched non-carcinoma tissues in uterine corpus endometrial carcinoma (UCEC), cholangiocarcinoma (CHOL), breast cancer (BRCA), head-neck squamous cell carcinoma (HNSC), esophageal carcinoma (ESCA), kidney renal papillary cell carcinoma (KIRP), kidney renal clear cell carcinoma (KIRC), lung adenocarcinoma (LUAD), LIHC, and stomach adenocarcinoma (STAD); whereas both were downregulated in kidney chromophobe. PD-1 was also upregulated in glioblastoma multiforme (GBM) and downregulated in thyroid cancer (THCA). CTLA4 expression was elevated in colon adenocarcinoma (COAD), lung squamous cell carcinoma (LUSC), and prostate adenocarcinoma (PRAD) and reduced in thymoma (THYM). The expression profiles of PD-1 and CTLA4 in TCGA cohorts are shown in Figures 1C,D, respectively, and PD-1 and CTLA4 gene expression matrices for the 33 cancer types in TCGA are shown in Supplementary Table 1.

**Figure 2:**
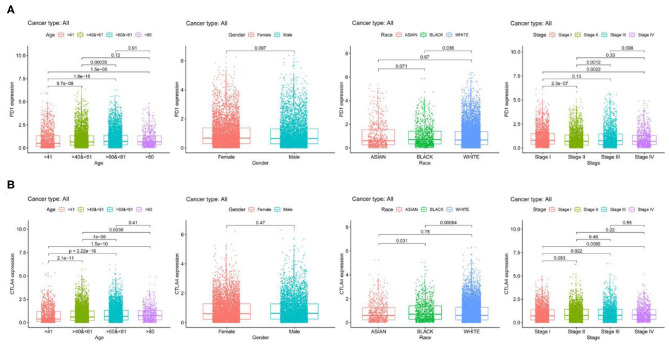
PD-1 and CTLA4 expression across 21 cancer types. **(A,B)** Boxplots of PD-1 **(A)** CTLA4 **(B)** levels.

We examined PD-1 and CTLA4 expression according to age, sex, race, and tumor stage and found that older patients had higher expressions of these genes than younger patients, while no differences were observed between sexes (Figure 2). Black patients had higher PD-1 and CTLA4 levels than Caucasian patients. PD-1 level was higher whereas CTLA4 level was lower in stage I disease compared to other stages.

### Association Between PD-1/CTLA4 Levels and Survival

**Figure 3:**
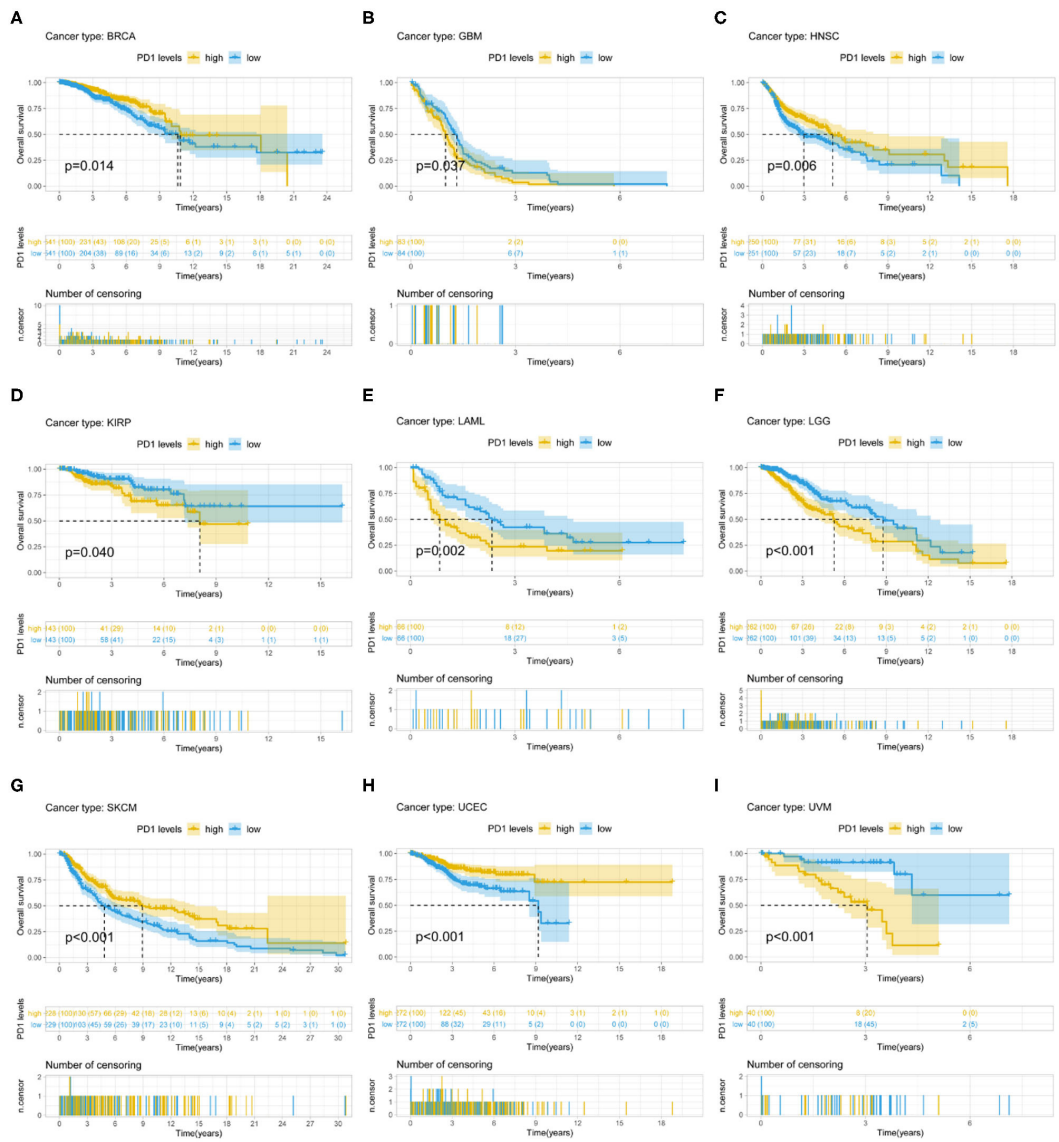
Association between PD-1 expression and OS. **(A–I)** Kaplan–Meier analysis of the association between PD-1 expression and OS.

PD-1 expression was identified by Cox regression analysis as a prognostic factor for OS in urothelial bladder carcinoma (BLCA), BRCA, cervical squamous cell carcinoma and endocervical adenocarcinoma (CESC), GBM, HNSC, KIRC, acute myeloid leukemia (LAML), STAD, UCEC, and uveal melanoma (UVM) (Table 1). The Kaplan–Meier survival analysis showed that subjects with higher PD-1 levels had shorter OS than those with lower levels in GBM (*P* = 0.037), KIRP (*P* = 0.040), LAML (*P* = 0.002), low-grade glioma (LGG) (*P* < 0.001), and UVM (*P* < 0.001). On the other hand, subjects with higher PD-1 levels had longer OS than those with lower levels in BRCA (*P* = 0.014), HNSC (*P* = 0.006), skin cutaneous melanoma (SKCM) (*P* < 0.001), and UCEC (*P* < 0.001) (Figures 3A–I).

**Table 1:** Univariate Cox regression analysis of the associations of PD-1 and CTLA4 expression with patient survival.

**Cancer**	**PD-1-OS**			**PD-1-PFI**			**CTLA4-OS**			**CTLA4-PFI**		
	**HR**	**HR (95% CI)**	***P*-value**	**HR**	**HR (95% CI)**	***P*-value**	**HR**	**HR (95% CI)**	***P*-value**	**HR**	**HR (95% CI)**	***P*-value**
ACC	1.338	0.670–2.675	0.409	0.675	0.281–1.626	0.381	1.305	0.477–3.571	0.605	0.605	0.201–1.825	0.372
BLCA	0.813	0.676–0.977	0.027	0.777	0.645–0.936	0.008	0.826	0.694–0.984	0.032	0.750	0.627–0.896	0.002
BRCA	0.753	0.595–0.953	0.018	0.688	0.538–0.879	0.003	0.845	0.681–1.049	0.127	0.831	0.672–1.028	0.088
CESC	0.683	0.514–0.907	0.008	0.686	0.516–0.912	0.010	0.652	0.482–0.882	0.006	0.581	0.423–0.799	0.001
CHOL	0.979	0.657–1.461	0.919	0.952	0.655–1.383	0.795	0.630	0.286–1.387	0.251	0.388	0.161–0.937	0.035
COAD	1.219	0.872–1.704	0.247	1.130	0.837–1.525	0.424	0.767	0.537–1.095	0.144	0.855	0.631–1.160	0.315
DLBC	0.807	0.461–1.413	0.454	1.203	0.745–1.942	0.451	0.972	0.569–1.660	0.918	1.113	0.701–1.767	0.649
ESCA	1.066	0.745–1.523	0.727	0.909	0.658–1.256	0.564	0.898	0.659–1.225	0.498	0.832	0.628–1.102	0.199
GBM	2.048	1.156–3.628	0.014	1.585	0.894–2.813	0.115	1.307	0.945–1.807	0.106	1.023	0.691–1.515	0.910
HNSC	0.764	0.638–0.914	0.003	0.814	0.679–0.974	0.025	0.725	0.606–0.868	<0.001	0.799	0.671–0.951	0.012
KICH	1.113	0.085–14.499	0.935	1.439	0.280–7.412	0.663	3.155	0.054–185.196	0.580	7.337	0.560–96.103	0.129
KIRC	1.210	1.064–1.375	0.004	1.130	0.995–1.284	0.060	1.619	1.292–2.028	<0.001	1.345	1.080–1.675	0.008
KIRP	1.568	1.241–1.981	<0.001	1.421	1.141–1.769	0.002	1.804	1.054–3.088	0.031	1.651	1.045–2.608	0.032
LAML	1.316	1.021–1.697	0.034	\	\	\	1.452	0.959–2.198	0.078	\	\	\
LGG	3.423	2.153–5.441	<0.001	2.381	1.578–3.594	<0.001	2.671	1.566–4.556	<0.001	3.365	2.057–5.505	<0.001
LIHC	1.009	0.818–1.245	0.935	0.884	0.732–1.069	0.203	1.008	0.740–1.374	0.959	0.918	0.709–1.188	0.516
LUAD	0.978	0.813–1.177	0.814	0.967	0.818–1.143	0.694	0.777	0.638–0.945	0.012	0.884	0.743–1.051	0.161
LUSC	1.007	0.846–1.199	0.934	0.997	0.814–1.220	0.975	1.007	0.837–1.210	0.943	0.947	0.765–1.173	0.617
MESO	1.001	0.781–1.282	0.995	0.983	0.728–1.327	0.912	1.078	0.804–1.446	0.615	0.859	0.572–1.289	0.463
OV	0.885	0.703–1.114	0.297	0.867	0.712–1.055	0.154	0.711	0.508–0.996	0.047	0.739	0.557–0.980	0.035
PAAD	0.952	0.695–1.304	0.759	1.028	0.792–1.334	0.835	0.970	0.715–1.316	0.846	1.034	0.795–1.345	0.803
PCPG	0.770	0.085–6.953	0.816	1.865	0.652–5.333	0.245	0.010	0.000–19.263	0.232	1.411	0.213–9.346	0.721
PRAD	0.728	0.155–3.422	0.687	1.356	0.929–1.980	0.115	0.571	0.109–2.993	0.507	1.358	0.986–1.870	0.061
READ	1.054	0.408–2.718	0.914	0.958	0.441–2.083	0.914	0.974	0.522–1.817	0.934	1.133	0.682–1.882	0.631
SARC	0.853	0.691–1.053	0.139	0.947	0.810–1.108	0.499	0.886	0.663–1.184	0.413	0.965	0.773–1.204	0.751
SKCM	0.764	0.683–0.855	<0.001	0.898	0.823–0.980	0.016	0.783	0.691–0.888	<0.001	0.907	0.823–1.000	0.050
STAD	0.794	0.644–0.978	0.030	0.942	0.768–1.155	0.565	0.775	0.624–0.962	0.021	0.875	0.705–1.085	0.223
TGCT	1.373	0.507–3.717	0.533	0.927	0.686–1.255	0.625	2.026	0.563–7.294	0.280	0.869	0.604–1.250	0.448
THCA	0.706	0.322–1.546	0.384	1.032	0.774–1.375	0.832	0.896	0.384–2.091	0.800	1.207	0.861–1.692	0.276
THYM	0.801	0.423–1.515	0.495	1.108	0.691–1.776	0.669	1.954	1.191–3.203	0.008	1.966	1.343–2.879	0.001
UCEC	0.684	0.530–0.882	0.003	0.709	0.572–0.879	0.002	0.592	0.412–0.852	0.005	0.650	0.483–0.874	0.004
UCS	1.185	0.740–1.899	0.479	0.853	0.524–1.388	0.522	1.073	0.577–1.995	0.824	0.840	0.469–1.503	0.557
UVM	1.905	1.289–2.816	0.001	1.506	1.007–2.253	0.046	3.299	1.299–8.379	0.012	2.088	0.745–5.849	0.161

*ACC, adrenocortical carcinoma; BLCA, bladder urothelial carcinoma; BRCA, breast invasive carcinoma; CESC, cervical squamous cell carcinoma and endocervical adenocarcinoma; CHOL, cholangiocarcinoma; COAD, colon adenocarcinoma; DLBC, lymphoid neoplasm diffuse large B-cell lymphoma; ESCA, esophageal carcinoma; GBM, glioblastoma multiforme; HNSC, head and neck squamous cell carcinoma; KICH, kidney chromophobe; KIRC, kidney renal clear cell carcinoma; KIRP, kidney renal papillary cell carcinoma; LAML, acute myeloid Leukemia; LGG, brain lower grade glioma; LIHC, liver hepatocellular carcinoma; LUAD, lung adenocarcinoma; LUSC, lung squamous cell carcinoma; MESO, Mesothelioma; OV, ovarian serous cystadenocarcinoma; PAAD, pancreatic adenocarcinoma; PCPG, pheochromocytoma and paraganglioma; PRAD, prostate adenocarcinoma; READ, Rectum adenocarcinoma; SARC, sarcoma; SKCM, skin cutaneous melanoma; STAD, stomach adenocarcinoma; TGCT, testicular germ cell tumors; THCA, thyroid carcinoma; THYM, thymoma; UCEC, uterine corpus endometrial carcinoma; UCS, uterine carcinosarcoma; UVM, uveal melanoma*.

PD-1 expression was also a prognostic factor for PFS in BLCA, BRCA, CESC, HNSC, KIRP, SKCM, UCEC, and UVM (Table 1). The Kaplan–Meier survival analysis showed that patients with higher PD-1 expression had shorter OS than those with lower expression in LGG (*P* < 0.001) and UVM (*P* = 0.025), whereas patients with higher PD-1 levels had longer OS than those with lower PD-1 levels in BRCA (*P* = 0.018), CESC (*P* = 0.047), SKCM (*P* = 0.047), and UCEC (*P* < 0.001) (Supplementary Figures 2A–F).

**Figure 4:**
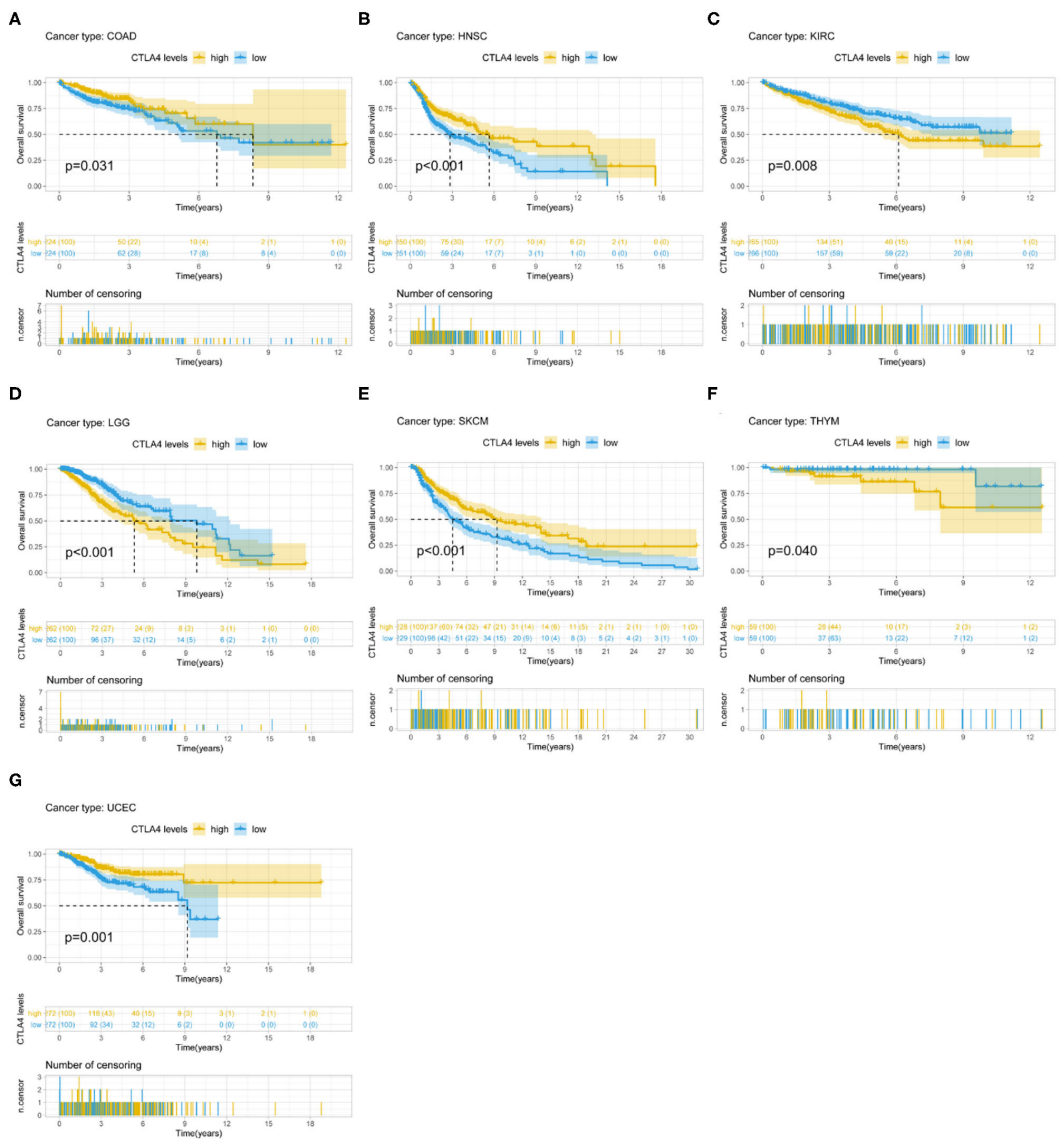
Association between CTLA4 expression and OS. **(A–G)** Kaplan–Meier analysis of the association between CTLA4 expression and OS.

CTLA4 expression was identified by Cox regression analysis as a prognostic factor for OS in BLCA, BRCA, CESC, GBM, HNSC, KIRC, LAML, STAD, UCEC, and UVM (Table 1). The Kaplan–Meier survival analysis showed that patients with higher CTLA4 expression had shorter OS than those with lower CTLA4 expression in KIRC (*P* = 0.008), LGG (*P* < 0.001), and THYM (*P* = 0.040). Meanwhile, patients with higher CTLA4 levels had longer OS than those with lower levels in COAD (*P* = 0.031), HNSC (*P* < 0.001), SKCM (*P* < 0.001), and UCEC (*P* = 0.001) (Figures 4A–G).

CTLA4 expression was also a prognostic factor for PFS in BLCA, CESC, CHOL, HNSC, KIRC, KIRP, ovarian serous cystadenocarcinoma (OV), THYM, and UCEC (Table 1). The Kaplan–Meier survival analysis showed that patients with higher CTLA4 expression had shorter OS than those with lower expression in KIRC (*P* = 0.021), LGG (*P* < 0.001), and THYM (*P* = 0.010), whereas patients with higher CTLA4 levels had longer OS than those with lower levels in BLCA (*P* = 0.011), HNSC (*P* = 0.004), and UCEC (*P* = 0.002) (Supplementary Figures 3A–F).

**Figure 5:**
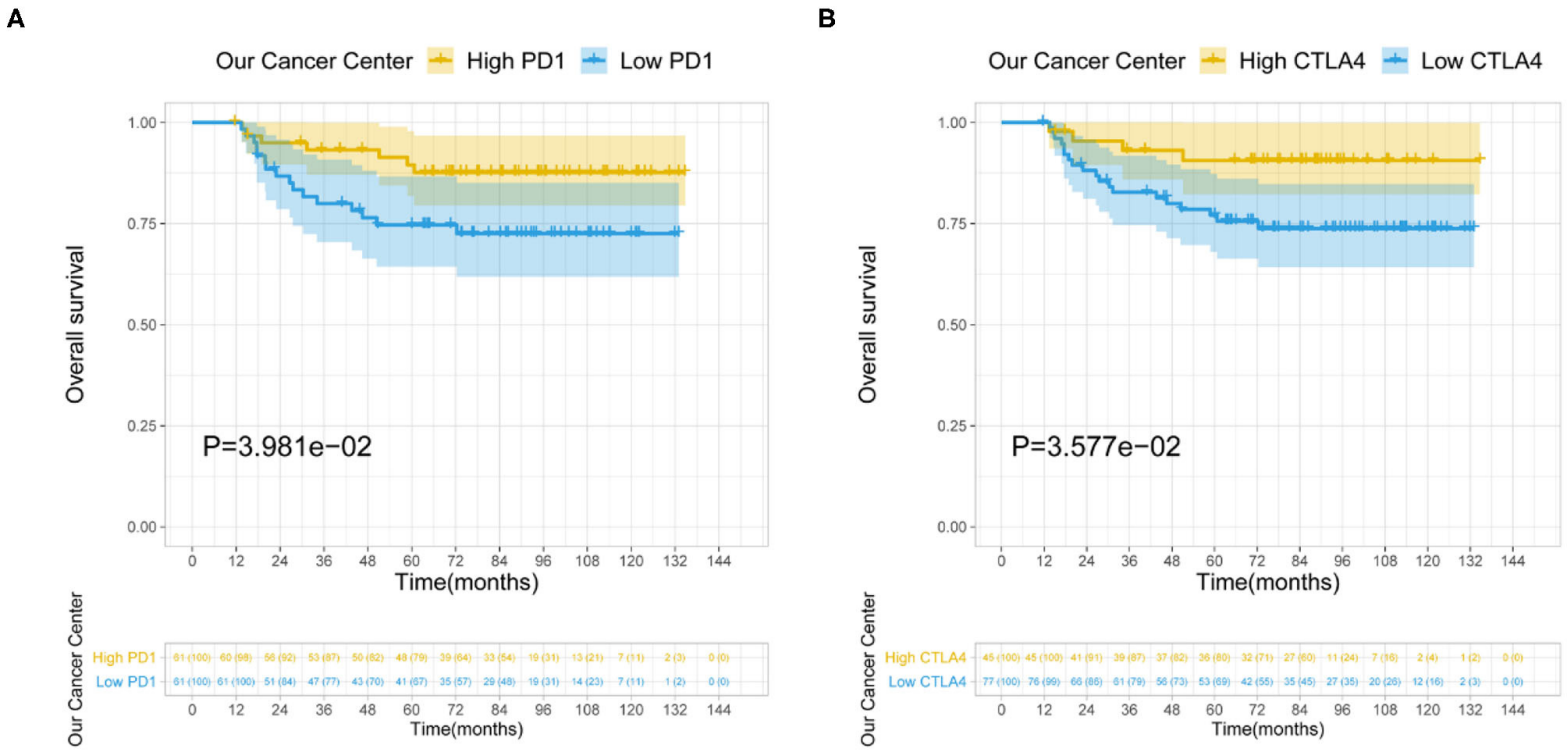
Relationship between serum PD-1/CTLA4 levels and prognosis of LIHC patients receiving CIK therapy. **(A,B)** Serum levels of PD-1 **(A)** and CTLA4 **(B)** are shown.

Among the LIHC patients at our hospital, the median serum PD-1 level was 82.9 μg/μl (range, 7.6–2,886.8); serum CTLA4 was undetectable in 77 patients (63.1%), and the maximum level was 88.6 μg/μl in the others (*n* = 45; 36.9%). The survival analysis showed that among LIHC patients who underwent CIK cell therapy, higher levels of PD-1 (*P* = 0.040) and CTLA4 (*P* = 0.036) were associated with longer OS (Figure 5).

### Relationship Between PD-1/CTLA4 Levels and Degree of Immune Cell Infiltration

**Figure 6:**
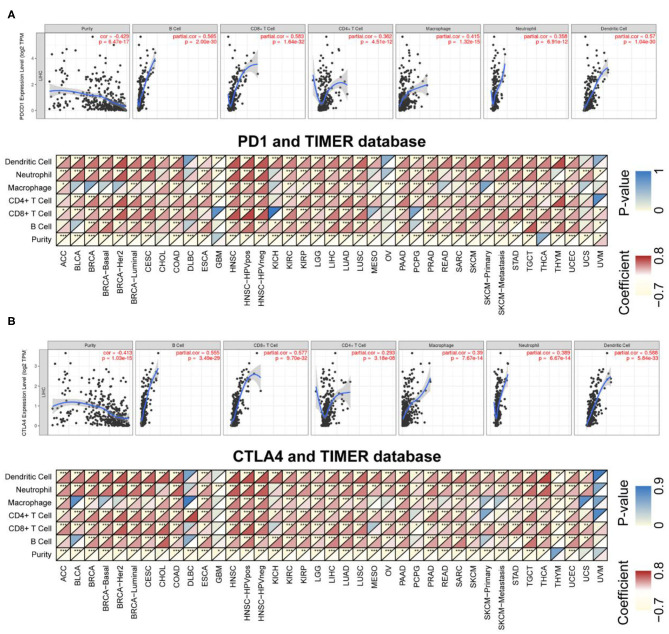
Correlation between PD-1/CTLA4 expression and cancer immunity in TIMER. **(A)**, Top Association between PD-1 level and degree of TIL infiltration in LIHC. (Bottom) TIMER prediction of the association between PD-1 level and degree of TIL infiltration in 33 cancer types. **(B)** TIMER prediction of the relationship between CTLA4 level and degree of TIL infiltration in LIHC and 33 cancer types. For each pair in **(A,B)**, the left top triangle is colored to represent the *P*-value, and the right bottom triangle is colored to represent the Spearman correlation coefficient. *P < 0.05, **P < 0.01, ***P < 0.001.

TILs are independent predictors of sentinel lymph node status as well as survival. We examined the correlation between PD-1/CTLA4 levels and the degree of immune cell infiltration in diverse cancer types in TIMER. PD-1 and CTLA4 levels were significantly associated with tumor purity in 35 and 36 cancer types, respectively. Additionally, PD-1 and CTLA4 levels were correlated with the degree of infiltration of CD4+ T cells in 33 and 33 cancer types, respectively; of B cells in 30 and 32 cancer types, respectively; of CD8+ T cells in 32 and 34 cancer types, respectively; of macrophages in 24 and 25 cancer types, respectively; of dendritic cells in 35 and 36 cancer types, respectively; and of neutrophils in 32 and 35 cancer types, respectively. The FDA granted the accelerated approval for the use of PD-1 in combination with CTLA4 for the treatment of LIHC (19). In this study, the association of the degree of immune infiltration with the levels of PD-1 and CTLA4 within LIHC is presented as an example in the top panels of Figures 6A,B. While the pan-cancer correlations of immune infiltration level with PD-1 and CTLA4 expression are present in the bottom panels of Figures 6A,B and Supplementary Table 2, respectively.

**Figure 7:**
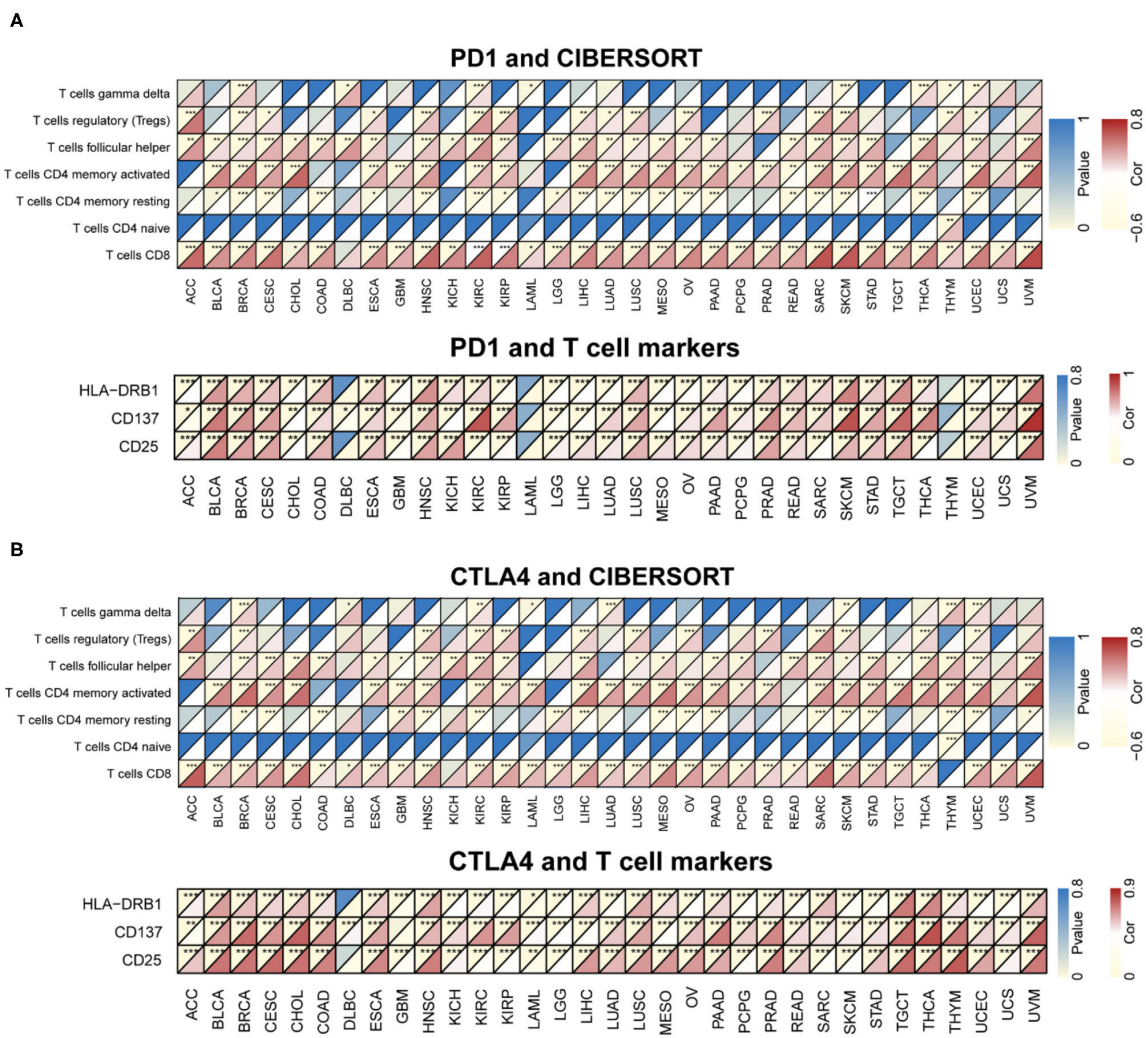
Correlation between PD-1 and CTLA4 expression and cancer immunity in CIBERSORT. **(A,B)** CIBERSORT prediction of the association between PD-1 **(A)** and CTLA4 **(B)** levels and degree of TIL infiltration in 33 cancer types. For each pair, the left top triangle is colored to represent the *P*-value, and the bottom right triangle is colored to represent the Spearman correlation coefficient. *P < 0.05, **P < 0.01, ***P < 0.001.

CIBERSORT was used to determine immunocyte profiles in all TCGA patients, and the correlation between 22 immunocytes and PD-1/CTLA4 expression was determined for the 33 cancer types in TCGA (Supplementary Table 3). PD-1 and CTLA4 were significantly correlated with CD8+ T cell but not CD4+ naïve T cell counts in most cancers (Figure 7). Additionally, the expression of T cell markers (CD25, CD137, and human leukocyte antigen DRB1) was correlated with PD-1 and CTLA4 levels.

**Figure 8:**
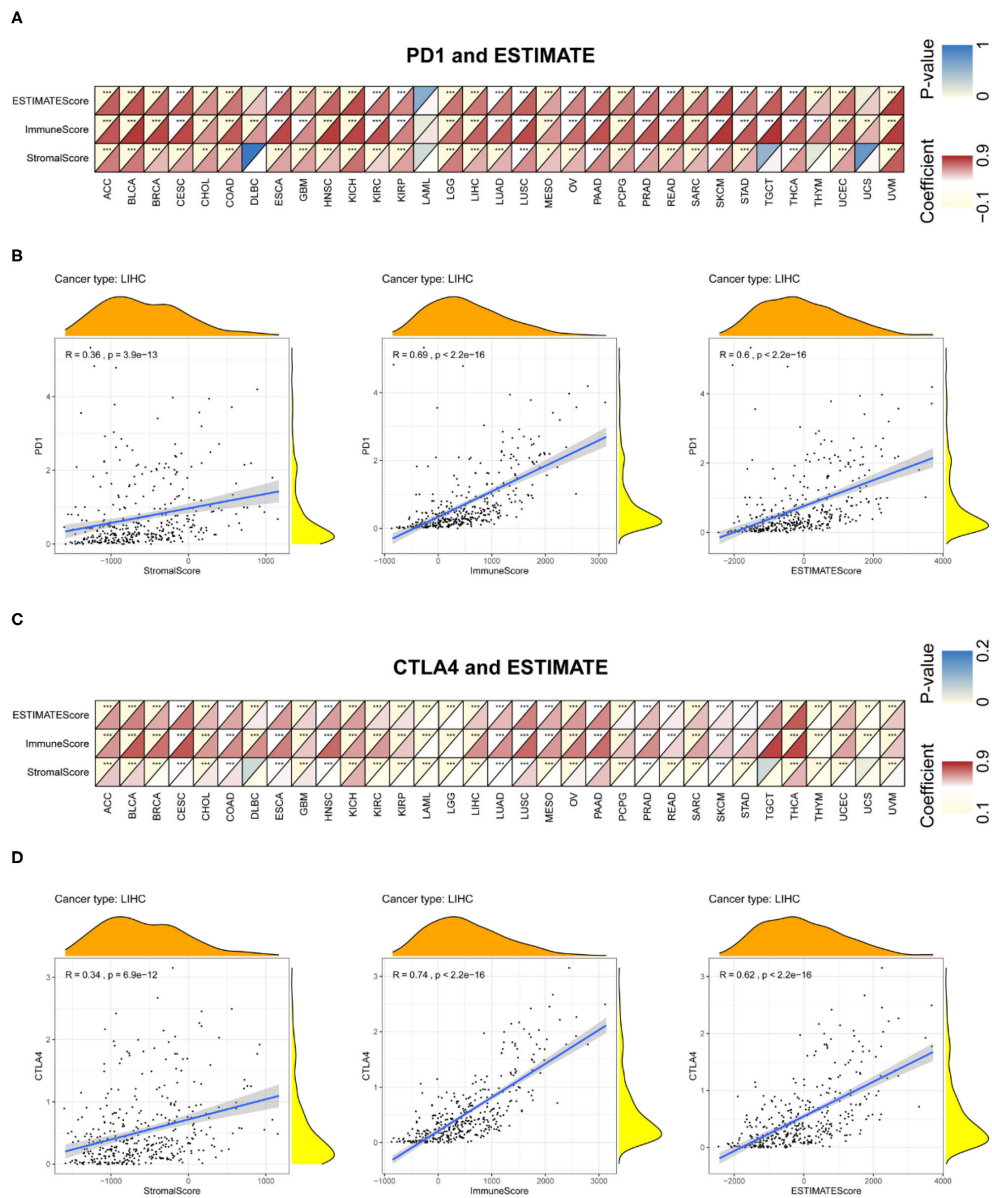
Correlation between PD-1 and CTLA4 expression and cancer immunity in ESTIMATE. ESTIMATE prediction of the relationship between PD-1 **(A,B)** and CTLA4 **(C,D)** levels and the degree of TIL infiltration in 33 cancer types **(A,C)** and LIHC **(B,D)**. For each pair, the top left triangle is colored to represent the *P*-value, and the bottom right triangle is colored to represent the Spearman correlation coefficient. *P < 0.05, **P < 0.01, ***P < 0.001.

Immune and stromal scores for tumor tissues were calculated using ESTIMATE; we then assessed the associations between these scores and PD-1 and CTLA4 expression (Figure 8 and Supplementary Table 4). Figures 8B,D exhibit the typical results in LIHC. The results showed that PD-1 and CTLA4 levels were significantly correlated with immune and stromal scores as well as estimate scores.

### Correlations Between PD-1/CTLA4 and Immune Cell Marker Expression

**Figure 9:**
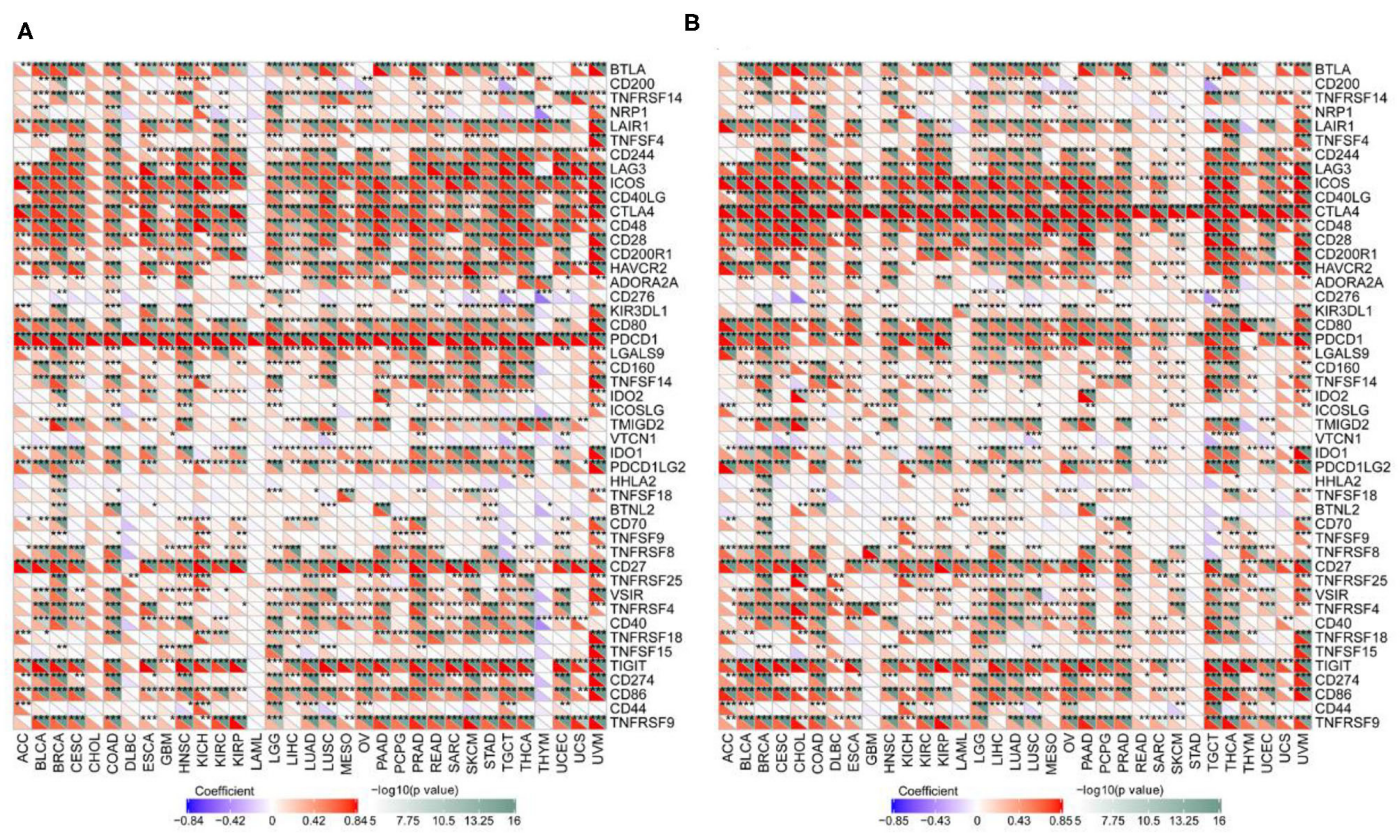
Correlation between PD-1 and CTLA4 levels and expression of immune markers. **(A,B)** Heatmap of the relationship between PD-1 **(A)** and CTLA4 **(B)** levels and expression T cell and other immunocyte markers in 33 cancer types. For each pair, the top right triangle is colored to represent the *P*-value, and the bottom left triangle is colored to represent the correlation coefficient. *P < 0.05, **P < 0.01, ***P < 0.001.

We examined the associations between TIL markers and PD-1/CTLA4 expression and found that PD-1 and CTLA4 levels were significantly correlated with the expression of T cell and other immunocyte markers (Figure 9), suggesting that both factors are involved in the regulation of the immune response to these cancers.

### Correlation Analysis of TMB, MSI, MMR, and DNMT Expression

**Figure 10:**
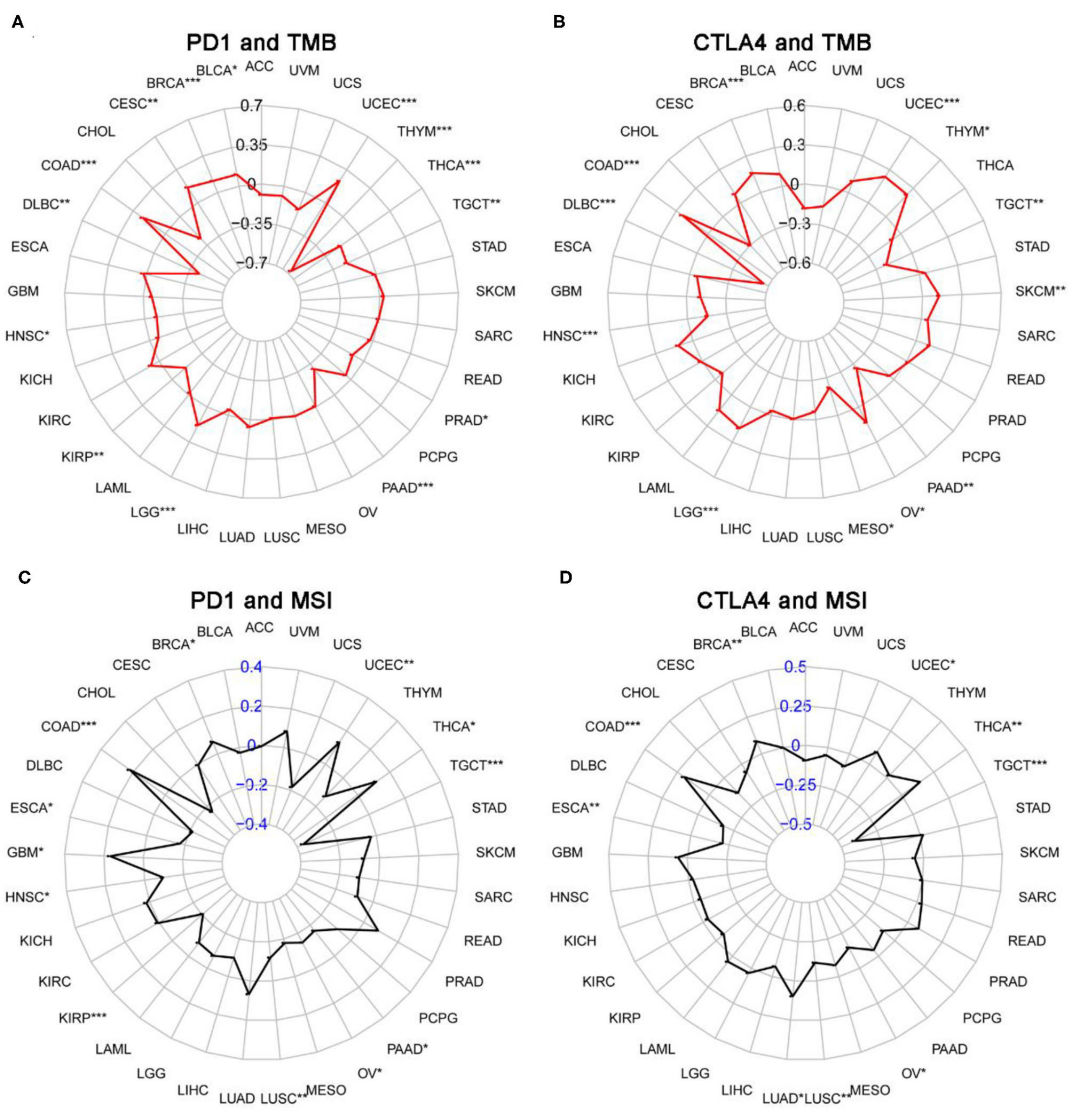
Correlation between PD-1 and CTLA4 expression and TMB and MSI. **(A,C)** Radar chart showing the correlation between PD-1 and TMB **(A)** and MSI **(C)** in 33 cancer types. Black and blue numbers represented the Spearman correlation coefficient. **(B,D)** Relationship between CTLA4 and TMB **(B)** and MSI **(D)**. *P < 0.05, **P < 0.01, ***P < 0.001.

**Figure 11:**
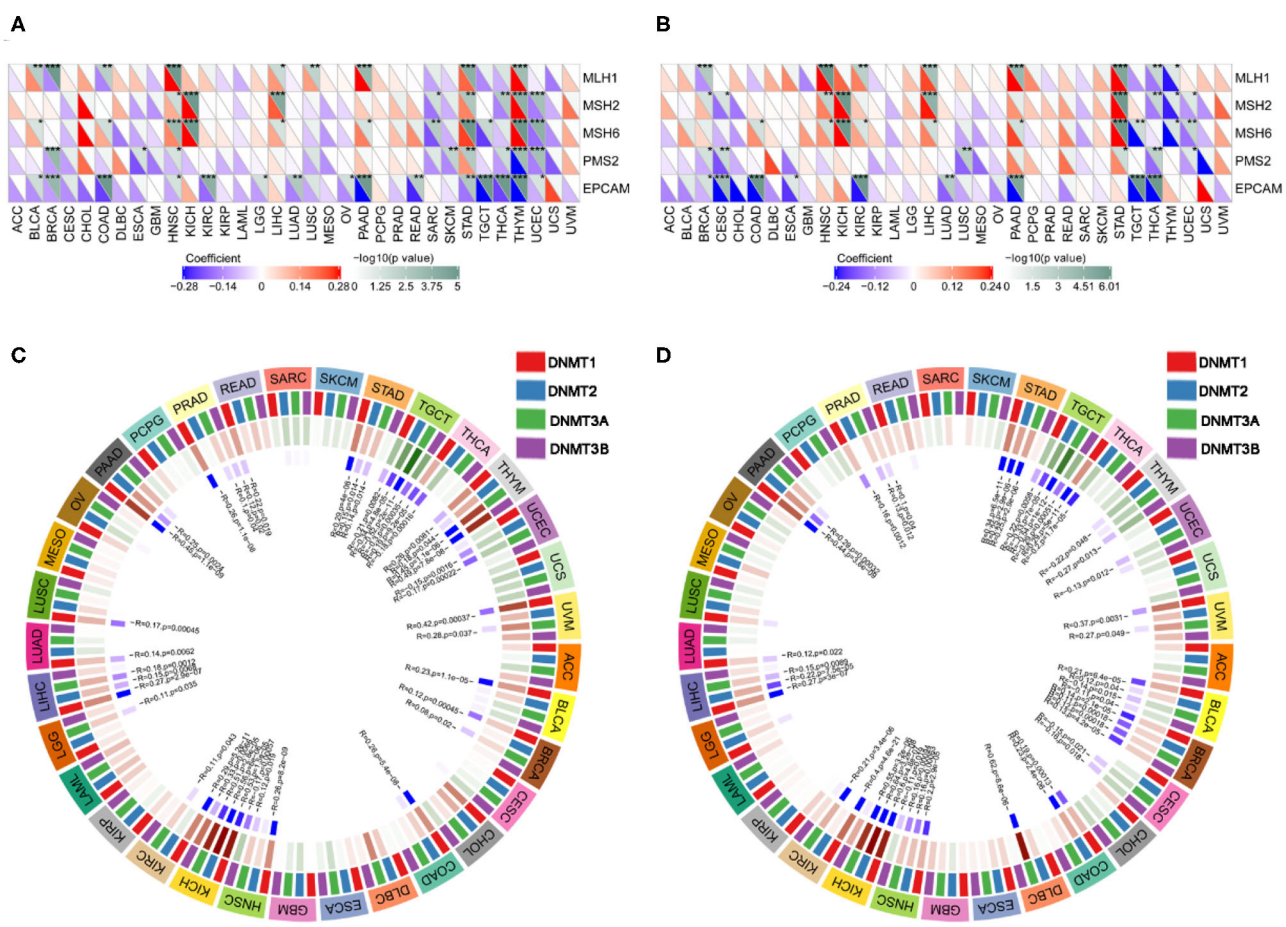
Correlation between PD-1 and CTLA4 expression and MMR and DNMT levels. **(A)** Heatmap of the correlation between PD-1 expression and MMR in 33 cancer types. For each pair, the top right triangle is colored to represent the *P*-value, and the bottom left triangle is colored to represent the Spearman correlation coefficient. **(B,D)** Relationship between CTLA4 level and MMR **(B)** and DNMT levels **(D)**. **(C)** Circle chart of the correlation between PD-1 and DNMT expression in 33 cancer types. The first outer ring represents cancer types; the second ring shows 4 DNMTs; the third ring shows correlation coefficients; the fourth ring is colored to represent *P*-values; and numbers in the inner ring are correlation coefficients and *P*-values.

We next examined the associations between PD-1/CTLA4 expression and TMB, MSI, MMR, and DNMT levels (Supplementary Table 5 and Figures 10, 11). PD-1 expression was correlated with TMB in BRCA, BLCA, COAD, CESC, HNSC, lymphoid neoplasm diffuse large b-cell lymphoma (DLBC), LGG, KIRP, PRAD, pancreatic adenocarcinoma (PAAD), testicular germ cell tumors (TGCT), THCA, THYM, and UCEC (Figure 10A); and CTLA4 expression was correlated with TMB in DLBC, BRCA, COAD, OV, HNSC, mesothelioma, LGG, SKCM, UCEC, PAAD, THYM, and TGCT (Figure 10B). PD-1 expression was correlated with MSI in COAD, BRCA, GBM, ESCA, OV, KIRP, HNSC, TGCT, LUSC, PAAD, THCA, and UCEC (Figure 10C); and CTLA4 expression was correlated with MSI in BRCA, COAD, ESCA, LUAD, LUSC, OV, TGCT, THCA, and UCEC (Figure 10D). Correlations between the expression of MMR genes (*MLH1, MSH2, MSH6, PMS2*, and *EPCAM*) and PD-1 and CTLA4 levels are shown in Figures 11A,B, respectively; and correlations between the expression of DNA methylation regulatory genes (*DNMT1, DNMT2, DNMT3A*, and *DNMT3B*) and PD-1 and CTLA4 levels are shown in Figures 11C,D, respectively.

### Functional Analysis

**Figure 12:**
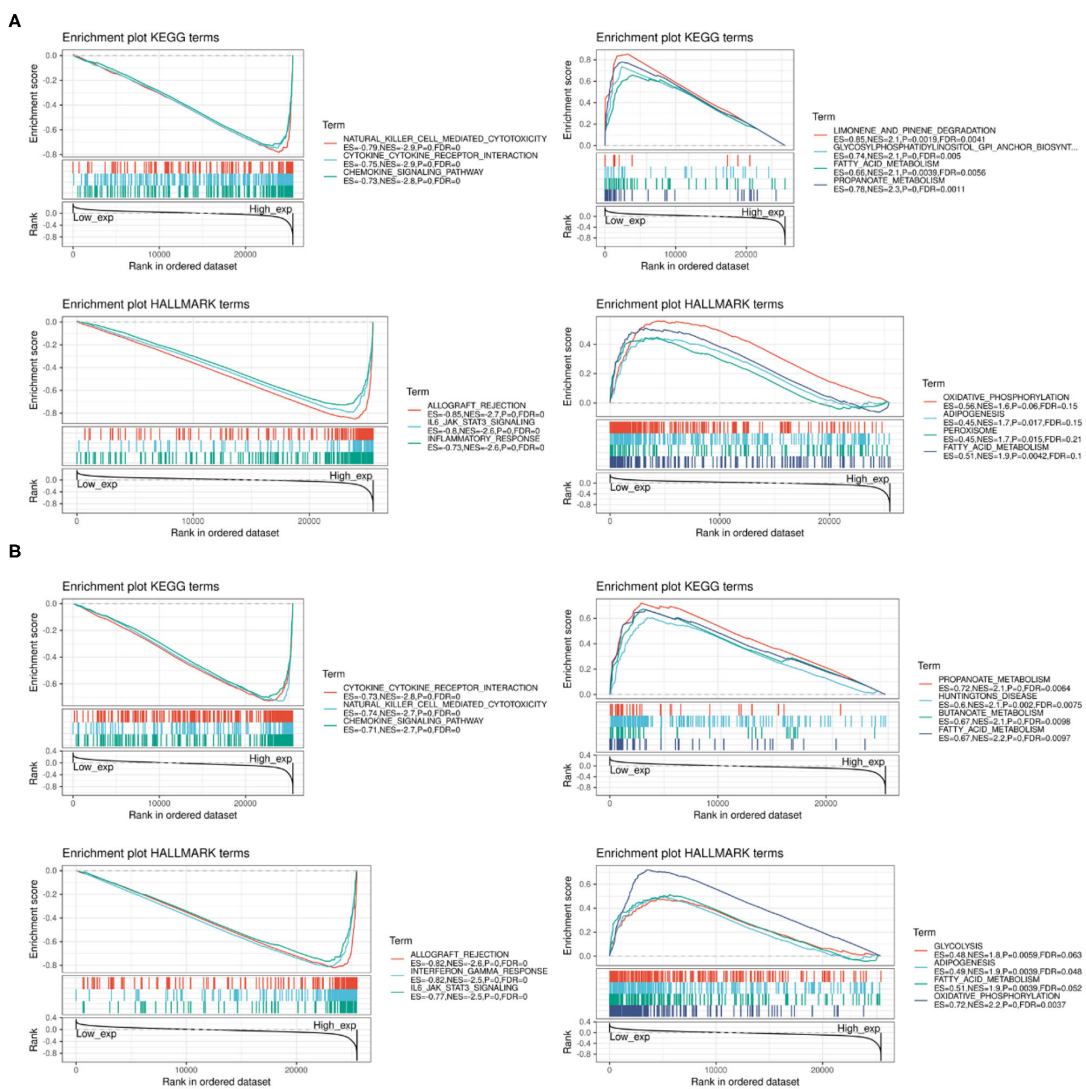
GSEA of top functional terms associated with PD-1 and CTLA4 expression. **(A,B)** Top KEGG and HALLMARK terms related to PD-1 **(A)** and CTLA4 **(B)**.

We performed GSEA to assess the biological significance of PD-1 and CTLA4 expression in different cancers. The functional Kyoto Encyclopedia of Genes and Genomes (KEGG) and Hallmark terms for PD-1 and CTLA4 are listed in Figures 12A,B, respectively.

## Discussion

Combination therapy with immune checkpoint inhibitors including anti–PD-1 and –CTLA4 antibodies shows greater therapeutic efficacy than the monotherapies in several cancers (31–35). In the present study, we used a comprehensive pan-cancer approach to evaluate the clinical significance of PD-1 and CTLA4 expression in a variety of cancers. Our results showed that PD-1 and CTLA4 expression varies across cancer types and that most cancers are characterized by PD-1 and CTLA4 mutations that lead to their abnormal expression, which can serve as a prognostic biomarker. Serum PD-1 and CTLA4 levels were survival predictor in LIHC patients receiving CIK therapy. Using TIMER, CIBERSORT, and ESTIMATE, we found that PD-1 and CTLA4 overexpression was associated with TIL infiltration, immune scores, and immune marker expression. Furthermore, PD-1 and CTLA4 levels were correlated with TMB, MSI, MMR, and the expression of DNMTs. We also identified KEGG and Hallmark terms that are associated with PD-1 and CTLA4 expression.

Identifying aberrantly expressed genes in tumors is important for the development of individualized treatments, which can improve therapeutic outcomes (36). Pan-cancer analyses can reveal the functional significance of PD-1 and CTLA4 in cancers (37, 38). Here we examined PD-1 and CTLA4 expression in a large and diverse set of samples from CCLE, which provides gene expression data for future experiments, and from TCGA, which provides genomic and survival data that may be useful for clinical investigations. Consistent with previous reports (39, 40), we found that PD-1 was more highly expressed in older cancer patients, indicating that checkpoint inhibitor treatment may more effective in this group. Additionally, Black patients had higher PD-1 and CTLA4 levels than Caucasian or Asian patients, suggesting better outcomes following immunotherapy.

Our results showed that PD-1 and CTLA4 are implicated in cancer immunity, as evidenced by the association between PD-1 and CTLA4 levels and the degree of infiltration of immunocytes in the TIMER and CIBERSORT analyses. The ESTIMATE method has been used to assess genomic data in various cancers, including the prediction of clinical outcomes in GBM and SKCM (41, 42). We determined immune and stromal scores based on TCGA data and found that PD-1 and CTLA4 levels were correlated with ESTIMATE scores as well as the expression of TIL markers.

Gene mutations are the major cause of cancer development (43), and specific mutations predict treatment response and prognosis (44, 45). TMB affects the generation of immunogenic peptides, thereby affecting patients' response to immune checkpoint inhibitor treatment (46, 47). Additionally, TMB and MSI reflect the production of new antibodies, with the latter being linked to increased TMB (48). In cervical squamous cell carcinoma and adrenocortical carcinoma, MSI-H was associated with an abnormally high mutation frequency (49). Thus, MSI is an important predictor of tumor development (29). MSI testing is recommended in the National Comprehensive Cancer Network guidelines for all colorectal cancer subtypes, as early detection of MSI-H can reduce mortality (50). Cancer cells with MMR deficiency (dMMR) can generate heterologous antigens that are recognized by T cells. PD-1 inhibitors are highly effective against MSI-H solid tumors (51); accordingly, the FDA has approved the use of the anti–PD-1 immunotherapy pembrolizumab for the treatment of MSI-H/dMMR solid tumors (51). Thus, TMB, MSI, and MMR can be used to predict therapeutic responses. In this study, we showed that PD-1 and CTLA4 levels were correlated with TMB and MSI in BRCA, COAD, TGCT, and UCEC. However, it remains to be determined whether the combination of PD-1 and CTLA4 inhibitors has greater efficacy than monotherapy in these cancers. Epigenetic modifications modulate gene expression and can be exploited by tumor cells to evade immune surveillance. A potential therapeutic strategy to circumvent this problem is to combine immune checkpoint and methylase inhibitors (30). PD-1 and CTLA4 have been implicated in various pathways related to immune function (52, 53). We carried out GSEA to identify clinically relevant pathways that may provide clues for future research. Taken together, our findings provided clues for the roles of PD-1 and CTLA4 in cancer immunity. However, these results should be interpreted with caution since checkpoint inhibitor treatment not analyzed in our work. And more experiments are needed to demonstrate our results, such as immunohistochemistry.

In conclusion, the results of our pan-cancer analysis indicate that PD-1 and CTLA4 are useful prognostic biomarkers in some cancer types. Importantly, we found that PD-1/CTLA4 expression is associated with cancer immunity. The integrative omics-based workflow in this study can serve as a basis for developing and testing hypotheses regarding novel targets in cancer treatment.

## Data Availability Statement

All datasets presented in this study are included in the article/Supplementary Material.

## Author Contributions

J-NL, WL, and Q-FC conceived of and designed the study. J-NL, X-SK, TH, WL, and Q-FC performed the literature search, generated the figures and tables, and wrote the manuscript. J-NL, TH, X-SK, and RW collected, analyzed the data, and critically reviewed the manuscript. WL and Q-FC supervised the study and reviewed the manuscript. All authors contributed to the article and approved the submitted version.

## Conflict of Interest

The authors declare that the research was conducted in the absence of any commercial or financial relationships that could be construed as a potential conflict of interest.

## Supplementary Material

Supplementary Figure 1 Overall study design.

Supplementary Figure 2 Association between PD-1 level and PFS. **(A–I)** Kaplan–Meier analysis of the association between PD-1 expression and PFS.

Supplementary Figure 3 Association between CTLA4 level and PFS. **(A–F)** Kaplan–Meier analysis of the association between CTLA4 expression and PFS.

Supplementary Table 1 PD-1 and CTLA4 expression across 33 cancers.

Supplementary Table 2 Associations between PD-1/CTLA4 expression levels and CD4+ and CD8+ T cell, B cell, dendritic cell, neutrophil, and macrophage counts based on TIMER.

Supplementary Table 3 Association between PD-1/CTLA4 expression levels and immunocyte marker expression as determined using CIBERSORT.

Supplementary Table 4 Associations between PD-1/CTLA4 expression levels and ESTIMATE scores.

Supplementary Table 5 Associations between PD-1/CTLA4 expression levels and TMB and MSI.

## References

[1] Bray F Ferlay J Soerjomataram I Siegel RL Torre LA Jemal A. Global cancer statistics 2018: GLOBOCAN estimates of incidence and mortality worldwide for 36 cancers in 185 countries. CA: Cancer J Clin. (2018) 68:394–424. 10.3322/caac.21492 30207593

[2] Cancer Genome Atlas Research N Weinstein JN Collisson EA Mills GB Shaw KR Ozenberger BA. The cancer genome atlas pan-Cancer analysis project. Nat Genet. (2013) 45:1113–20. 10.1038/ng.2764 24071849 PMC3919969

[3] Li W Chen QF Huang T Shen L Huang ZL Wu P. Profiles of m(6)A RNA methylation regulators for the prognosis of hepatocellular carcinoma. Oncol Lett. (2020) 19:3296–306. 10.3892/ol.2020.11435 32256825 PMC7074306

[4] Huang ZL Li W Chen QF Wu PH Shen LJ. Eight key long non-coding RNAs predict hepatitis virus positive hepatocellular carcinoma as prognostic targets. World J Gastroint Oncol. (2019) 11:983–97. 10.4251/wjgo.v11.i11.983 31798779 PMC6883184

[5] Cancer Cell Line Encyclopedia C, Genomics of Drug Sensitivity in Cancer C. Pharmacogenomic agreement between two cancer cell line data sets. Nature. (2015) 528:84–7. 10.1038/nature15736 26570998 PMC6343827

[6] Ye Y Hu Q Chen H Liang K Yuan Y Xiang Y. Characterization of hypoxia-associated molecular features to aid hypoxia-targeted therapy. Nat Metabo. (2019) 1:431–44. 10.1038/s42255-019-0045-8 31984309 PMC6980239

[7] Barger CJ Branick C Chee L Karpf AR. Pan-cancer analyses reveal genomic features of FOXM1 overexpression in cancer. Cancers. (2019) 11:251. 10.3390/cancers11020251 30795624 PMC6406812

[8] Schaub FX Dhankani V Berger AC Trivedi M Richardson AB Shaw R. Pan-cancer alterations of the MYC oncogene and its proximal network across the cancer genome atlas. Cell Syst. (2018) 6:282–300 e2. 29596783 10.1016/j.cels.2018.03.003 PMC5892207

[9] Danaher P Warren S Lu R Samayoa J Sullivan A Pekker I. Pan-cancer adaptive immune resistance as defined by the tumor inflammation signature (TIS): results from The cancer genome atlas (TCGA). J Immunother Cancer. (2018) 6:63. 10.1186/s40425-018-0367-1 29929551 PMC6013904

[10] Vaddepally RK Kharel P Pandey R Garje R Chandra AB. Review of indications of FDA-approved immune checkpoint inhibitors per NCCN guidelines with the level of evidence. Cancers. (2020) 12:738. 10.3390/cancers12030738 32245016 PMC7140028

[11] Chen L Flies DB. Molecular mechanisms of T cell co-stimulation and co-inhibition. Nat Rev Immunol. (2013) 13:227–42. 10.1038/nri3405 23470321 PMC3786574

[12] Schadendorf D Hodi FS Robert C Weber JS Margolin K Hamid O Pooled Analysis of long-term survival data from phase II and phase III trials of ipilimumab in unresectable or metastatic melanoma. J Clin Oncol. (2015) 33:1889–94. 10.1200/JCO.2014.56.2736 25667295 PMC5089162

[13] Larkin J Minor D D'Angelo S Neyns B Smylie M Miller WH. Overall survival in patients with advanced melanoma who received nivolumab versus investigator's choice chemotherapy in checkmate 037: a randomized, controlled, open-label phase III trial. J Clin Oncol. (2018) 36:383–90. 10.1200/JCO.2016.71.8023 28671856 PMC6804912

[14] Larkin J Chiarion-Sileni V Gonzalez R Grob JJ Rutkowski P Lao CD. Five-year survival with combined nivolumab and ipilimumab in advanced melanoma. N Engl J Med. (2019) 381:1535–46. 10.1056/NEJMoa1910836 31562797

[15] Motzer RJ Tannir NM McDermott DF Aren Frontera O Melichar B Choueiri TK. Nivolumab plus Ipilimumab versus sunitinib in advanced renal-cell carcinoma. N Engl J Med. (2018) 378:1277–90. 10.1056/NEJMoa1712126 29562145 PMC5972549

[16] Cella D Grunwald V Escudier B Hammers HJ George S Nathan P. Patient-reported outcomes of patients with advanced renal cell carcinoma treated with nivolumab plus ipilimumab versus sunitinib (CheckMate 214): a randomised, phase 3 trial. Lancet Oncol. (2019) 20:297–310. 10.1016/S1470-2045(18)30778-2 30658932 PMC6701190

[17] Motzer RJ Rini BI McDermott DF Aren Frontera O Hammers HJ Carducci MA. Nivolumab plus ipilimumab versus sunitinib in first-line treatment for advanced renal cell carcinoma: extended follow-up of efficacy and safety results from a randomised, controlled, phase 3 trial. Lancet Oncol. (2019) 20:1370–85. 10.1016/S1470-2045(19)30413-9 31427204 PMC7497870

[18] Hellmann MD Paz-Ares L Bernabe Caro R Zurawski B Kim SW Carcereny Costa E. Nivolumab plus Ipilimumab in advanced non-small-cell lung cancer. N Engl J Med. (2019) 381:2020–31. 10.1056/NEJMoa1910231 31562796

[19] Wright K. FDA approves nivolumab plus ipilimumab for the treatment of advanced HCC. Oncology. (2020) 34:693606. 32293694

[20] Li T Fan J Wang B Traugh N Chen Q Liu JS. TIMER: A web server for comprehensive analysis of tumor-infiltrating immune cells. Cancer Res. (2017) 77:e108–10. 10.1158/0008-5472.CAN-17-0307 29092952 PMC6042652

[21] Li B Severson E Pignon JC Zhao H Li T Novak J. Comprehensive analyses of tumor immunity: implications for cancer immunotherapy. Genome Biol. (2016) 17:174. 10.1186/s13059-016-1028-7 27549193 PMC4993001

[22] Chen QF Li W Wu PH Shen LJ Huang ZL. Significance of tumor-infiltrating immunocytes for predicting prognosis of hepatitis B virus-related hepatocellular carcinoma. World J Gastroenterol. (2019) 25:5266–82. 10.3748/wjg.v25.i35.5266 31558872 PMC6761238

[23] Yoshihara K Shahmoradgoli M Martinez E Vegesna R Kim H Torres-Garcia W. Inferring tumour purity and stromal and immune cell admixture from expression data. Nat Commun. (2013) 4:2612. 10.1038/ncomms3612 24113773 PMC3826632

[24] Siemers NO Holloway JL Chang H Chasalow SD Ross-MacDonald PB Voliva CF. Genome-wide association analysis identifies genetic correlates of immune infiltrates in solid tumors. PLoS ONE. (2017) 12:e0179726. 10.1371/journal.pone.0179726 28749946 PMC5531551

[25] Danaher P Warren S Dennis L D'Amico L White A Disis ML. Gene expression markers of tumor infiltrating leukocytes. J Immunother Cancer. (2017) 5:18. 10.1186/s40425-017-0215-8 28239471 PMC5319024

[26] Pan JH Zhou H Cooper L Huang JL Zhu SB Zhao XX. LAYN is a prognostic biomarker and correlated with immune infiltrates in gastric and colon cancers. Front Immunol. (2019) 10:6. 10.3389/fimmu.2019.00006 30761122 PMC6362421

[27] Krieger T Pearson I Bell J Doherty J Robbins P. Targeted literature review on use of tumor mutational burden status and programmed cell death ligand 1 expression to predict outcomes of checkpoint inhibitor treatment. Diagnos Pathol. (2020) 15:6. 10.1186/s13000-020-0927-9 32000815 PMC6990470

[28] Li K Luo H Huang L Luo H Zhu X. Microsatellite instability: a review of what the oncologist should know. Cancer Cell Int. (2020) 20:16. 10.1186/s12935-019-1091-8 31956294 PMC6958913

[29] Dan H Zhang S Zhou Y Guan Q. DNA methyltransferase inhibitors: catalysts for antitumour immune responses. OncoTargets Ther. (2019) 12:10903–16. 10.2147/OTT.S217767 31849494 PMC6913319

[30] Antonia SJ Lopez-Martin JA Bendell J Ott PA Taylor M Eder JP. Nivolumab alone and nivolumab plus ipilimumab in recurrent small-cell lung cancer (CheckMate 032): a multicentre, open-label, phase 1/2 trial. Lancet Oncol. (2016) 17:883–95. 10.1016/S1470-2045(16)30098-5 27269741

[31] Hammers HJ Plimack ER Infante JR Rini BI McDermott DF Lewis LD. Safety and efficacy of nivolumab in combination with ipilimumab in metastatic renal cell carcinoma: the checkmate 016 study. J Clini Oncol. (2017) 35:3851–8. 10.1200/JCO.2016.72.1985 28678668 PMC7587408

[32] Hellmann MD Rizvi NA Goldman JW Gettinger SN Borghaei H Brahmer JR. Nivolumab plus ipilimumab as first-line treatment for advanced non-small-cell lung cancer (CheckMate 012): results of an open-label, phase 1, multicohort study. Lancet Oncol. (2017) 18:31–41. 10.1016/S1470-2045(16)30624-6 27932067 PMC5476941

[33] Larkin J Chiarion-Sileni V Gonzalez R Grob JJ Cowey CL Lao CD Combined nivolumab and ipilimumab or monotherapy in untreated melanoma. N Engl J Med. (2015) 373:23–34. 10.1056/NEJMc1509660 26027431 PMC5698905

[34] Wolchok JD Kluger H Callahan MK Postow MA Rizvi NA Lesokhin AM. Nivolumab plus ipilimumab in advanced melanoma. N Engl J Med. (2013) 369:122–33. 10.1056/NEJMoa1302369 23724867 PMC5698004

[35] Andre F Mardis E Salm M Soria JC Siu LL Swanton C. Prioritizing targets for precision cancer medicine. Ann Oncol. (2014) 25:2295–303. 10.1093/annonc/mdu478 25344359

[36] Cao Z Zhang S. An integrative and comparative study of pan-cancer transcriptomes reveals distinct cancer common and specific signatures. Sci Rep. (2016) 6:33398. 10.1038/srep33398 27633916 PMC5025752

[37] Cava C Bertoli G Colaprico A Olsen C Bontempi G Castiglioni I. Integration of multiple networks and pathways identifies cancer driver genes in pan-cancer analysis. BMC Genom. (2018) 19:25. 10.1186/s12864-017-4423-x 29304754 PMC5756345

[38] Jeske SS Schuler PJ Doescher J Theodoraki MN Laban S Brunner C. Age-related changes in T lymphocytes of patients with head and neck squamous cell carcinoma. Immun Ageing. (2020) 17:3. 10.1186/s12979-020-0174-7 32082401 PMC7017629

[39] Kasanen H Hernberg M Makela S Bruck O Juteau S Kohtamaki L. Age-associated changes in the immune system may influence the response to anti-PD1 therapy in metastatic melanoma patients. Cancer Immunol Immunother. (2020) 69:717–30. 10.1007/s00262-020-02497-9 32036449 PMC7183505

[40] Jia D Li S Li D Xue H Yang D Liu Y. Mining TCGA database for genes of prognostic value in glioblastoma microenvironment. Aging. (2018) 10:592–605. 10.18632/aging.101415 29676997 PMC5940130

[41] Yang S Liu T Nan H Wang Y Chen H Zhang X. Comprehensive analysis of prognostic immune-related genes in the tumor microenvironment of cutaneous melanoma. J Cell Physiol. (2020) 235:1025–35. 10.1002/jcp.29018 31240705

[42] Martincorena I Campbell PJ. Somatic mutation in cancer and normal cells. Science. (2015) 349:1483–9. 10.1126/science.aab4082 26404825

[43] Sanz-Garcia E Argiles G Elez E Tabernero J. BRAF mutant colorectal cancer: prognosis, treatment, and new perspectives. Ann Oncol. (2017) 28:2648–57. 10.1093/annonc/mdx401 29045527

[44] Allegra CJ Rumble RB Hamilton SR Mangu PB Roach N Hantel A. Extended RAS gene mutation testing in metastatic colorectal carcinoma to predict response to anti-epidermal growth factor receptor monoclonal antibody therapy: American society of clinical oncology provisional clinical opinion update 2015. J Clin Oncol. (2016) 34:179–85. 10.1200/JCO.2015.63.9674 26438111

[45] Wu HX Wang ZX Zhao Q Chen DL He MM Yang LP. Tumor mutational and indel burden: a systematic pan-cancer evaluation as prognostic biomarkers. Ann Translat Med. (2019) 7:640. 10.21037/atm.2019.10.116 31930041 PMC6944566

[46] Havel JJ Chowell D Chan TA. The evolving landscape of biomarkers for checkpoint inhibitor immunotherapy. Nat Rev Cancer. (2019) 19:133–50. 10.1038/s41568-019-0116-x 30755690 PMC6705396

[47] Chalmers ZR Connelly CF Fabrizio D Gay L Ali SM Ennis R. Analysis of 100,000 human cancer genomes reveals the landscape of tumor mutational burden. Genome Med. (2017) 9:34. 10.1186/s13073-017-0424-2 28420421 PMC5395719

[48] Bonneville R Krook MA Kautto EA Miya J Wing MR Chen HZ. Landscape of microsatellite instability across 39 cancer types. JCO Preci Oncol. (2017) 2017. 10.1200/PO.17.00073 29850653 PMC5972025

[49] Benson AB 3rd Venook AP Cederquist L Chan E Chen YJ Cooper HS. Colon cancer, version 1.2017, NCCN clinical practice guidelines in oncology. J Natl Comprehen Cancer Netw. (2017) 15:370–98. 10.6004/jnccn.2017.0036 28275037

[50] Diaz LA Jr Le DT. PD-1 blockade in tumors with mismatch-repair deficiency. N Engl J Med. (2015) 373:1979. 10.1056/NEJMc1510353 26559582

[51] Yu Y. Molecular classification and precision therapy of cancer: immune checkpoint inhibitors. Front Med. (2018) 12:229–35. 10.1007/s11684-017-0581-0 29209918

[52] Mannick JB Del Giudice G Lattanzi M Valiante NM Praestgaard J Huang B. mTOR inhibition improves immune function in the elderly. Sci Trans Med. (2014) 6:268ra179. 10.1126/scitranslmed.3009892 25540326

[53] Bagherzadeh Yazdchi S Witalis M Meli AP Leung J Li X Panneton V. Hippo pathway kinase Mst1 is required for long-lived humoral immunity. J Immunol. (2019) 202:69–78. 10.4049/jimmunol.1701407 30478091 PMC6310088

