# Supplementary figures and images for: Clinical Implications of Aberrant PD-1 and CTLA4 Expression for Cancer Immunity and Prognosis: A Pan-Cancer Study
Source: Front Immunol. 2020 Sep 10;11:2048. doi: 10.3389/fimmu.2020.02048 (PMC7539667; doi:10.3389/fimmu.2020.02048)

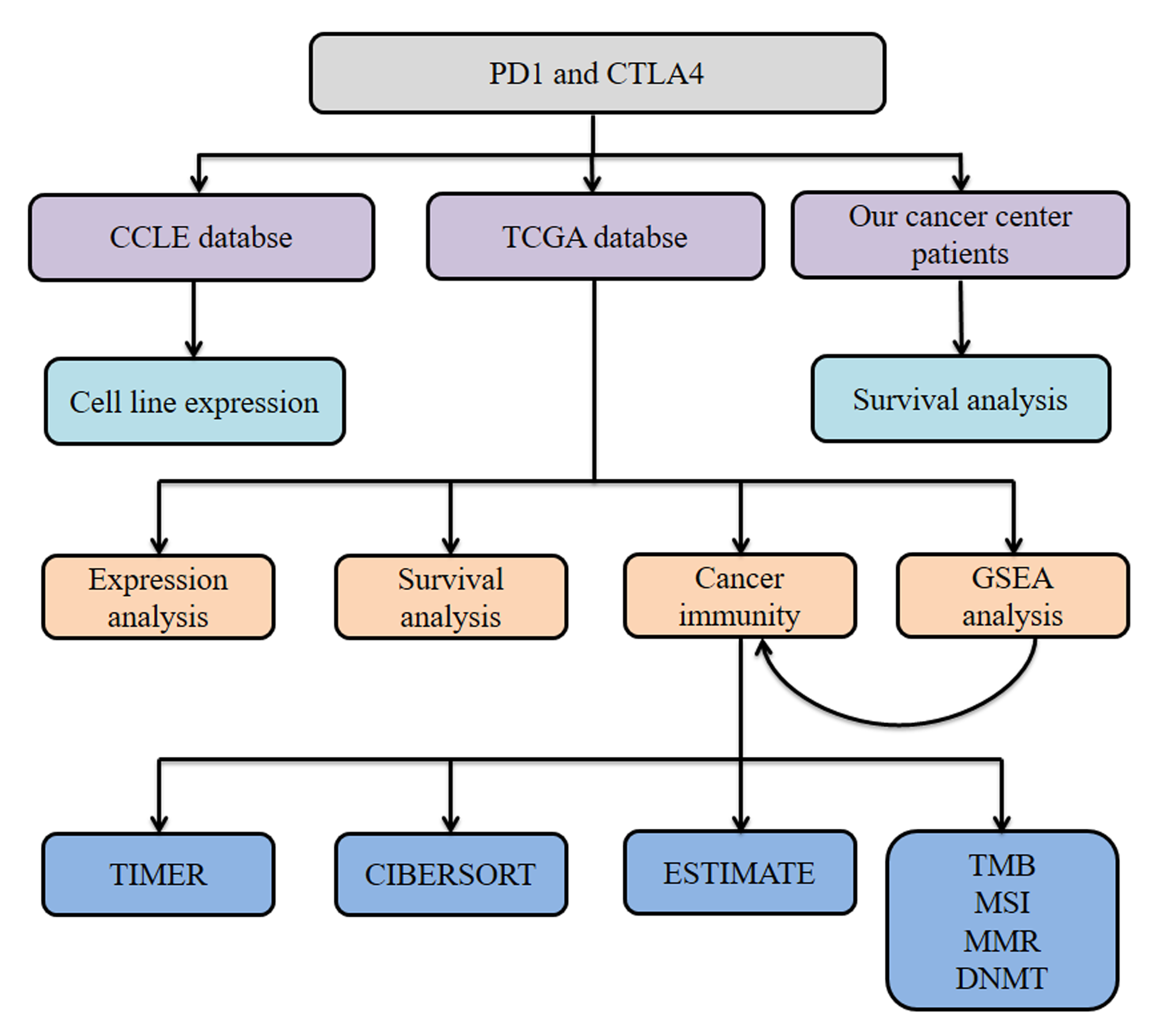

Supplement: Supplementary Figure 1 — Overall study design. [file Image_1.TIF]

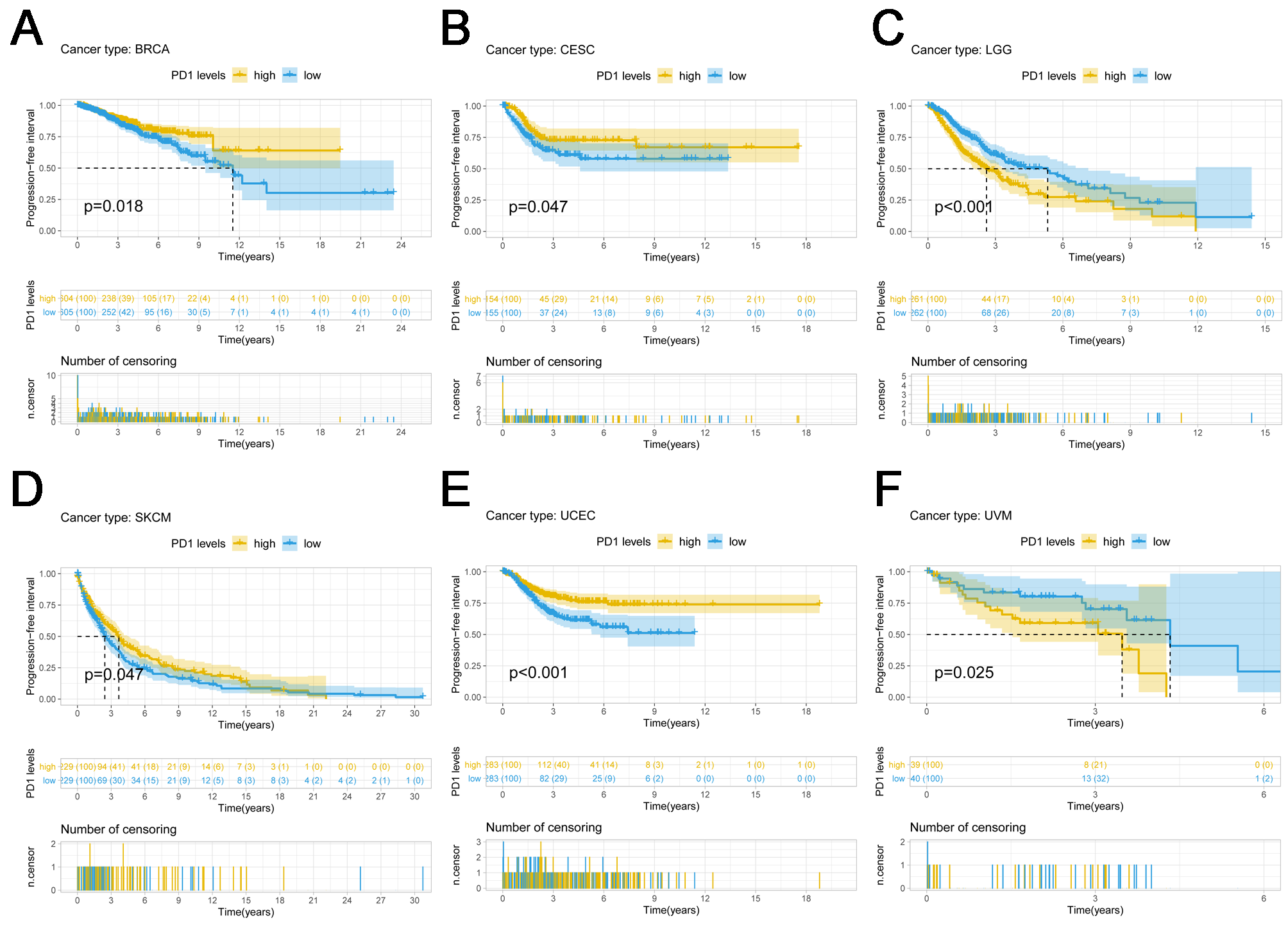

Supplement: Supplementary Figure 2 — Association between PD-1 level and PFS. (A–I) Kaplan–Meier analysis of the association between PD-1 expression and PFS. [file Image_2.TIF]

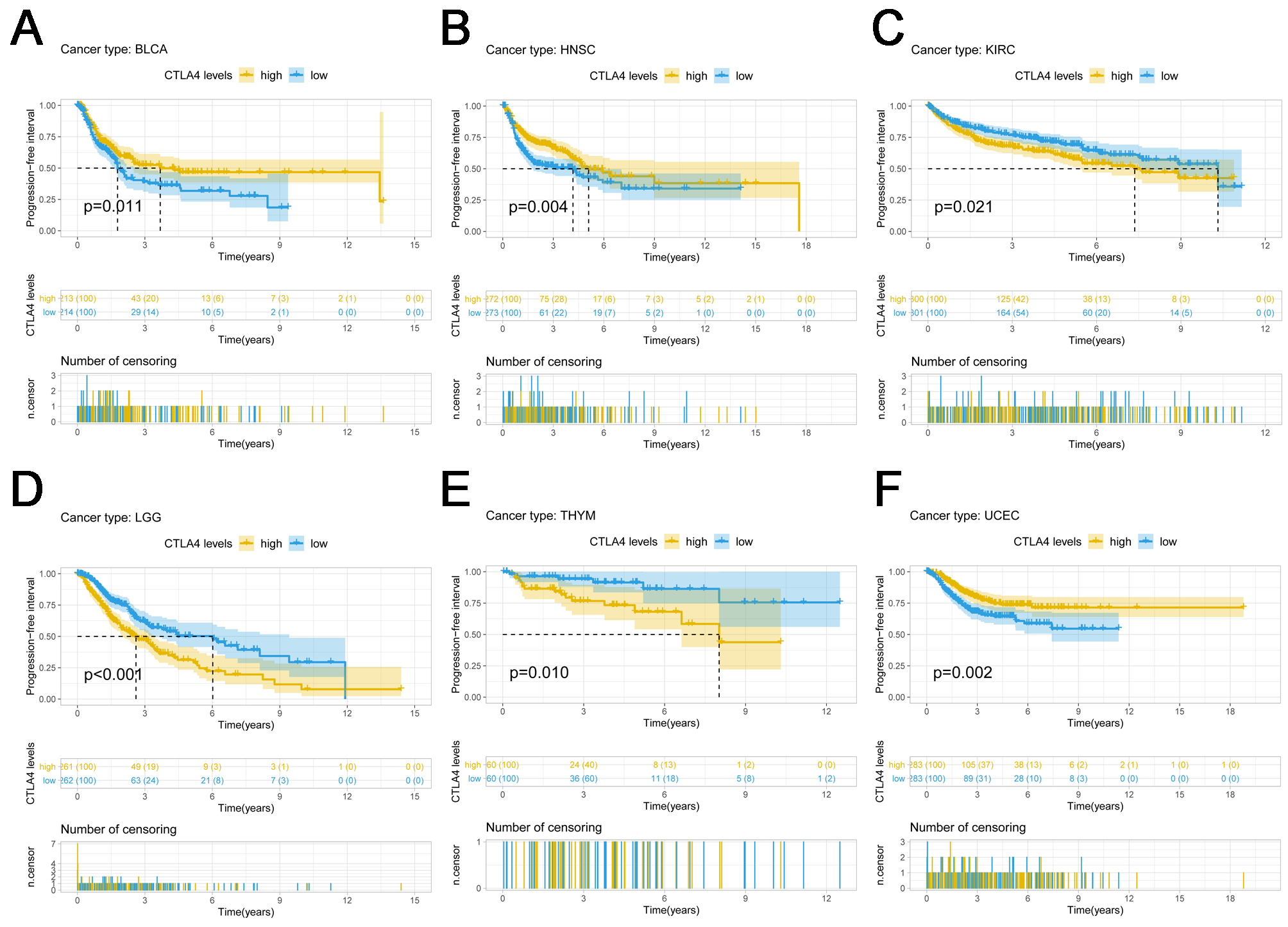

Supplement: Supplementary Figure 3 — Association between CTLA4 level and PFS. (A–F) Kaplan–Meier analysis of the association between CTLA4 expression and PFS. [file Image_3.TIF]
